# Clinical Features of Breast Cancer Patients with Human T-Cell Lymphotropic Virus Type-1 Infection

**DOI:** 10.31557/APJCP.2019.20.6.1909

**Published:** 2019

**Authors:** Munetsugu Hirata, Yoshiaki Shinden, Ayako Nagata, Yuki Nomoto, Hazuki Saho, Akihiro Nakajo, Takaaki Arigami, Hiroshi Kurahara, Kosei Maemura, Shoji Natsugoe, Yuko Kijima

**Affiliations:** 1 *Department of Breast Surgery, Fujita Medical University, 1-98 Dengakugakubo, Kutsukakecho, Toyoake, Aichi 470-1192, *; 2 *Department of Digestive Surgery, Breast and Thyroid Surgery, Kagoshima University Graduate School of Medical and Dental Sciences, 8-35-1, Sakuragaoka, Kagoshima 890-8520, Japan. *

**Keywords:** HTLV-1, breast cancer, clinicopathological factors

## Abstract

**Background:**

Human T-cell lymphotropic virus type 1 (HTLV-1) is a retrovirus that causes adult T-cell leukemia/lymphoma (ATL), an aggressive form of T-cell malignancy. The relationship between HTLV-1 infection and cancer progression is controversial. HTLV-1 encodes oncogenic protein TAX1 and it is hypothesized that HTLV-1 infection is associated with breast cancer progression. In this study, we evaluated the relationship between HTLV-1 infection and clinicopathological factors in breast cancer patients.

**Methods:**

We retrospectively analyzed 610 patients with primary breast cancer who underwent surgical treatment without preoperative chemotherapy at Kagoshima University Hospital between January 2001 and January 2015.

**Results:**

When patients with and without HTLV-1 infection were compared, no differences in clinicopathological factors were observed, except for age. Disease-free survival and overall survival rates did not differ between groups.

**Conclusions:**

HTLV-1–positive patients were significantly older than HTLV-1–negative patients. It was supposed to be due to the fact that the HTLV-1 infection rate is decreasing. Any effect of HTLV-1 infection on breast cancer progression appears to be negligibly small.

## Introduction

Human T-cell lymphotropic virus type 1 (HTLV-1) is a retrovirus that causes an aggressive form of T-cell malignancy called adult T-cell leukemia/lymphoma (ATL) (Arisawa et al., 2006). The association between HTLV-1 infection and cancer occurrence is controversial. HTLV-1–infected patients are reported to be at significantly high risk of developing liver cancer and lymphomas other than ATL (Arisawa et al., 2006; Tanaka et al., 2016). However, a lower incidence of HTLV-1 in gastric cancer patients compared to the general population suggests that HTLV-1 infection reduces the risk of gastric cancer (Hirata et al., 2007; Matsumoto et al., 2008; Tahaei et al., 2011). To the best of our knowledge, no reports have demonstrated a significant difference in breast cancer incidence between HTLV-1–infected patients and uninfected patients. 

Tax1, a protein encoded by HTLV-1, has demonstrated oncogenic properties via the Ras-Raf-MEK-ERK signaling pathway in breast cancer cell lines (Song et al., 2009). Thus, HTLV-1 infection has been hypothesized to be associated with breast cancer progression. In this study, we evaluated the relationship between HTLV-1 infection and clinicopathological factors in breast cancer patients.

## Materials and Methods

*Patients*

We enrolled 686 patients with primary breast cancer who underwent curative surgical treatment without preoperative chemotherapy at Kagoshima University Hospital, Kagoshima, Japan between January 2001 and January 2015. During this period, HTLV-1 infection status was checked as part of the preoperative routine examination. Diagnostic method for HTLV-1 infection was chemiluminescence immunoassay against HTLV-1 antigen. Patients with recurrent breast cancer and those for whom HTLV-1 infection status was not checked were excluded. As a result, 610 patients were included in the present analysis. Clinicopathological information was collected retrospectively using medical records, and the seventh edition of the TNM classification was used for staging. Adjuvant therapy was selected for each patient based on the National Comprehensive Cancer Network guidelines. 

*Statistical analysis*

Differences in clinicopathological factors between groups were evaluated using analysis of variance for continuous variables and the Pearson chi-squared test for categorical variables. Disease-free survival (DFS) and overall survival (OS) were measured from the time of first surgery until the date of death or last follow-up. Survival curves were calculated using the Kaplan-Meier method, and statistical significance between groups was assessed using the log-rank test. Statistical analysis was performed using JMP Pro, version 12.1.0 for Mac OS (SAS Institute Japan Ltd., Tokyo, Japan). P values <0.05 were considered statistically significant.

**Figure 1:**
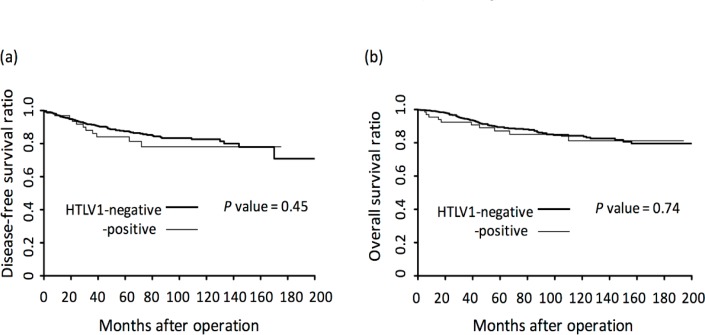
Kaplan-Meier Survival Curves of Breast Cancer Patients with and without HTLV-1 Infection. (a), Disease-free survival curve. HTLV-1 negative patients (bold line) and positive patients (thin line) were compared. (b), Overall survival curve

**Table 1:** Clinicopathological Factors of Enrolled Patients

N	610
Mean age, years (± S.D.)	60.4 ± 13.4
HTLV-1 infection, n (%)	
Positive	66 (11%)
Negative	544 (89%)
T factor, n	
Tis	78
T1	262
T2	175
T3	21
T4	74
Lymph node metastasis, n	
N0	416
N1	109
N2	27
N3	58
Stage, n	
0	78
I	223
II	201
III	93
IV	15
Estrogen receptor status, n	
Positive	480
Negative	123
NA	7
Progesterone receptor status, n	
Positive	403
Negative	201
NA	6
HER2 status, n	
Positive	89
Negative	450
NA*	71
Ki67 index, n	
<20%	107
≥20%	95
NA*	408
Recurrence, n (%)	
Yes	84 (14%)
-	511 (86%)
Death, n (%)	
Yes	85 (14%)
-	525 (86%)

*HER2 and Ki67 expression were not evaluated in some patients with ducal carcinoma in situ

**Table 2:** Comparison of Clinicopathological Factors between HTLV-1–Positive and Negative Patients

HTLV-1 infection			
	Positive	Negative	p value
n (%)	66 (11%)	544 (89%)	
Mean age, years	66.7	59.7	<0.0001
T factor, n			
Tis-1	39	301	0.56
T2-4	27	243	
Lymph node metastasis, n			
Negative	48	369	0.42
Positive	18	175	
Stage, n			
0-I	35	266	0.52
II-IV	31	278	
Nuclear grade, n			
1	28	204	0.76
2, 3	25	199	
NA	3	141	
Estrogen receptor status, n			
Positive	57	424	0.15
Negative	9	114	
NA	0	3	
Progesterone receptor status, n			
Positive	42	361	0.59
Negative	24	177	
NA	0	6	
HER2 expression status, n			
Positive	9	80	0.63
Negative	53	397	
NA	4	67	
Ki67 index, n			
< 20%	16	91	0.095
≥20%	7	88	
NA	43	365	
Recurrence, n			
Yes	11	73	0.53
-	55	456	
Death, n			
Yes	10	75	0.76
-	56	470	

## Results

*Patient characteristics*

Clinicopathological characteristics of enrolled patients are shown in Table 1. The mean age was 60.4 years, and 11% (n=66) of patients were HTLV-1–positive. No patient had ATL within the observation period. Immunohistochemical analysis was not performed in a small number of patients. Additionally, HER2 and Ki67 expression were not evaluated in some patients with ducal carcinoma in situ. The recurrence rate was 14% (n=85) among patients with stage 0-III disease, and 14% of patients (n=85) died during the observation period.

*Comparison of clinicopathological characteristics between patients with and without HTLV-1 infection*

When patients with and without HTLV-1 infection were compared, no differences in clinicopathological factors were observed, except for age (Table 2); HTLV-1–positive patients were significantly older than HTLV-1–negative patients. Rates of recurrence and death were not different (Table 2), and DFS and OS did not differ between groups (Figure 1).

## Discussion

HTLV-1 is a retrovirus that causes ATL, an aggressive form of T-cell malignancy (Arisawa et al., 2006). HTLV-1 is transmitted from mother to infant via breast milk and is known to be prevalent in several specific areas in the world, including Southwestern Japan (Arisawa et al., 2006). It was estimated that there were 10-20 millions HTLV-1 infected individuals in the world (de The and Bomford, 1993). In Japan, HTLV-1 infection has been extensively studied for many years, and infected individuals was estimated to be around 1.2 million (Tajima, 1990). The geographic distribution of HTLV-1 carriers is quite uneven in Japan and the greatest prevalence is observed in Southwestern Japan (Gessain et al., 2012). Our hospital located at Kagoshima in Southwestern Japan, and Kagoshima was reported to have highest HTLV-1 seropositive women rate in the world (Gessain et al., 2012). So, we routinely checked HTLV-1 infection status for all preoperative patients for a period of time. In the present cohort, 11% of patients had HTLV-1 infection. Although this infection rate is relatively high, it is compatible with previously reported rates in our geographic region. To the best of our knowledge, no reports of a significant association between HTLV-1 infection and breast cancer occurrence have been published. We did not have access to data from healthy controls to compare with the patients from the present cohort, so we could not analyze the association between HTLV-1 infection and breast cancer incidence. Rather, in this study we aimed to evaluate the effect of HTLV-1 infection on clinicopathological factors following breast cancer diagnosis. No significant differences were observed between HTLV-1–positive and –negative patients, except for age: the age at diagnosis was significantly higher among HTLV-1–positive patients compared HTLV-1–negative patients. This may be due in part to the fact that the HTLV-1 infection rate is decreasing. Overall, Satake et al. (2012) reported that the number of HTLV-1 carriers has decreased 10% over the past 2 decades (1980s to 2000s), so the age distribution of carriers has trended toward older patients (Satake et al., 2012).

HTLV-1 encodes Tax1, which was originally identified as a transcriptional activator that plays a central role in the immortalization of infected T-cells (Fujii et al., 2009). Tax1 has been reported to promote cancer cell proliferation via the Ras-Raf-MEK-ERK signaling pathway (Song et al., 2009). In addition, Tax1 sensitizes breast cancer cells to malignant transformation induced by environmental carcinogens through the inhibition of estrogen-induced ERα-mediated BRCA1 expression (Shukrun et al., 2014). Thus, we hypothesized that HTLV-1 infection is associated with breast cancer progression. However, no significant differences in tumor size, lymph node metastasis, stage, or other factors were observed between HTLV-1–positive and –negative patients. Furthermore, with respect to prognosis, no significant differences in recurrence or OS were observed. Any effect of HTLV-1 infection on breast cancer progression therefore appears to be negligibly small.

## Ethical Issue

This study was approved by the institutional review board of Kagoshima University Hospital. And for this type of study formal consent is not required.

## Conflict of interest

There is no financial relationships or other interests associated with this manuscript, which might be construed as constituting a conflict of interest. 
